# Interpersonal climate and moral conduct in competitive sport: A dual-route model with serial mediation

**DOI:** 10.1371/journal.pone.0339778

**Published:** 2025-12-31

**Authors:** Ziqi Wang, Taoming Liu, Siyu Hong, Wenjun Wang, Fenglin Wang

**Affiliations:** 1 Graduate School of Education, Shandong Sport University, Jinan, China; 2 School of Sports Training, Wuhan Sports University, Wuhan, China; 3 Department of General Education, Shandong First Medical University, Tai’an, China; Guangxi Normal University, CHINA

## Abstract

**Background:**

To investigate how coaching leadership styles influence athletes’ moral conduct, this study explored the direct and indirect effects of democratic and autocratic leadership behaviors on prosocial and antisocial behaviors in sport.

**Method:**

A cross-sectional survey was conducted among 1,239 competitive athletes from multiple sports, measuring leadership perception, goal orientation, and moral disengagement.

**Key results:**

Democratic leadership significantly predicted higher prosocial behavior and lower antisocial tendencies, while autocratic leadership showed the opposite pattern.

**Implications:**

These effects were mediated by athletes’ task and ego orientation, as well as their level of moral disengagement. The findings highlight the broader relevance of ethical, knowledge-based leadership in cultivating sustainable behavioral outcomes in sports. These results support social cognitive and moral disengagement theory by revealing how internal cognitive-motivational factors transmit leadership influence. The study contributes to both sport psychology and leadership literature by identifying key mediating mechanisms and clarifying the behavioral implications of different leadership approaches. Practically, within the context of Chinese elite sport, the findings suggest that fostering more democratic leadership styles may be associated with more ethical conduct and support athletes’ longer-term development, although cross-cultural and longitudinal research is needed before drawing broader causal conclusions.

## 1. Introduction

The ethical conduct of athletes has garnered increasing academic and institutional attention due to its critical role in promoting fair play, maintaining team cohesion, and preserving the social credibility of competitive sports [1,2]. However, in high-performance contexts where competitive pressure and performance outcomes are heavily emphasized, unethical behaviors such as intentional fouls, verbal aggression, and simulation still occur. These behaviors not only undermine interpersonal trust and team dynamics but also negatively affect public perception of athletes and sports organizations [3,4].

In sport psychology, moral behavior is commonly divided into prosocial behavior—voluntary actions that benefit others (e.g., helping an injured opponent or supporting teammates)—and antisocial behavior, referring to actions that harm or disadvantage others (e.g., cheating, aggression) [5–7]. Prosocial behaviors contribute to enjoyment, commitment, and team performance, while antisocial behaviors are linked to increased emotional exhaustion, team conflict, and diminished outcomes [8–10].

Researchers have identified both external social-contextual and internal psychological factors as important predictors of moral behavior in sport. Among social factors, coaching leadership style is a critical influence. Democratic and autonomy-supportive coaching behaviors have been associated with higher levels of prosocial behavior, whereas authoritarian or controlling leadership styles have been shown to foster antisocial tendencies [11,12]. Despite this, empirical research on the impact of coaching leadership on athlete behavior remains limited in non-Western contexts, especially in China [13,14].

At the psychological level, moral disengagement—also referred to in Chinese research as sport moral shirking—has been shown to facilitate antisocial behavior by allowing individuals to rationalize unethical conduct without experiencing guilt or self-condemnation. Previous studies have found that moral disengagement positively predicts antisocial behavior and, in some cases, negatively predicts prosocial behavior [15,16].

In addition, goal orientation, typically conceptualized as task orientation (emphasizing self-improvement) and ego orientation (emphasizing outperforming others), has been closely associated with athletes’ moral cognition and behavior. Ego orientation is positively related to both moral disengagement and antisocial behavior, while task orientation has been linked to increased prosocial behavior and moral accountability [17,18]. These findings suggest a complex interaction between leadership style, goal orientation, and moral disengagement in determining athletes’ moral decision-making and behavioral tendencies.

Despite substantial theoretical progress, integrated tests remain scarce in Chinese competitive sport. Few studies examine how coaching leadership and the two psychological mediators jointly shape athletes’ moral conduct. Independent and serial mediation pathways are rarely tested. Clarifying these pathways in a non-WEIRD, high-performance context addresses a persistent gap and tests whether leadership effects propagate simultaneously through motivational (task/ego orientation) and moral (disengagement) mechanisms. Practically, evidence from Chinese elite sport can inform coach education and governance standards aimed at curbing antisocial conduct without compromising performance.

The cultural distinctives of Chinese competitive sport are shaped by a centralized, selection-based pipeline that structures athlete development from sport schools to provincial teams and ultimately national teams. This system fosters hierarchical coach-athlete relationships intertwined with collectivist norms, which likely amplify the importance of normative comparison and the acceptance of controlling supervision. At the same time, these dynamics coexist with culturally specific benevolent practices in coach-athlete interactions. Recent research in China has identified three key interpersonal coaching styles—benevolent, autonomy-supportive, and controlling—which both overlap with and extend beyond traditional Western democratic-autocratic distinctions [19]. In the present study, we retained the democratic–autocratic distinction for two main reasons. First, it is the predominant framework in the international literature on leadership, motivation, and moral behaviour in sport, which allows our findings to be compared more directly with prior work and to be integrated with established SCT- and AGT-based models. Second, the benevolent, autonomy-supportive, and controlling styles identified in Chinese settings map conceptually onto more democratic versus more controlling/autocratic climates, so that testing the dual-route model with DL and AL provides an initial bridge between indigenous insights and the broader leadership–morality tradition, and we retain the democratic–autocratic (DL/AL) distinction as the analytic backbone for four reasons. First, DL/AL is anchored in the Leadership Scale for Sports (LSS), whose subscales have been repeatedly validated and enable psychometric comparability across studies, including Chinese samples [20,21]. Second, our hypotheses and mediation tests map directly onto established linkages between coaching style, goal orientations, and moral disengagement in sport, supporting a coherent test of both motivational and moral–cognitive routes [22]. Third, the DL/AL framing yields a parsimonious specification for testing independent and sequential mediations without inflating model complexity [23]. Fourth, positioning DL/AL as the core lens does not preclude cultural specificity; rather, we view the indigenous triad of benevolent, autonomy-supportive, and controlling styles as a meaningful extension to be evaluated with measurement-invariance and moderated-mediation designs [24].

Accordingly, the present paper tests a dual-route model under the DL/AL framework while explicitly acknowledging the relevance of indigenous interpersonal styles in Chinese high-performance sport, which we identify as a priority for cross-cultural generalization in the limitations and future-research sections.

Emerging findings from Chinese samples further reveal that coach-created motivational climates influence antisocial behavior in athletes through the mechanism of moral disengagement [25], while abusive supervision within this system is driven by unique antecedents distinct from those observed in Western contexts [26].

To address these gaps, the present study investigates how democratic and authoritarian coaching leadership behaviors influence athletes’ prosocial and antisocial behaviors in sport. Furthermore, it examines whether these relationships are mediated by goal orientation and moral disengagement, both independently and in a sequential pathway. By integrating insights from ethical leadership theory and sport morality research, this study aims to contribute both theoretical and practical knowledge for promoting ethical behavior in athletic contexts.

This study is guided by the following research questions:

1. How do democratic and authoritarian coaching leadership behaviors affect athletes’ prosocial and antisocial behaviors?
2. Do goal orientation (task and ego) and moral disengagement serve as mediators in these relationships?
3. Are there sequential mediating effects from goal orientation to moral disengagement in influencing athletes’ moral behavior?

This study addresses key gaps in the literature by making three incremental contributions to understanding leadership and morality in sport. First, it introduces a dual-route model that links democratic and autocratic coaching styles to prosocial and antisocial behavior through both motivational mechanisms (task and ego orientations) and a moral-cognitive mechanism (moral disengagement), analyzed simultaneously rather than separately. This integrated approach goes beyond previous research, which has typically examined achievement-goal or moral-disengagement pathways in isolation, and clarifies their combined and unique roles in shaping athletes’ moral behavior. Second, by differentiating independent from sequential mediation, the study identifies the ego-orientation → moral-disengagement → antisocial-behavior pathway as the most robust route, whereas task orientation plays a less prominent sequential role in high-performance settings. This nuanced pattern refines existing goal-orientation theories by specifying how different goal orientations feed into moral disengagement. Third, the study tests this model in a large, multi-sport sample of Chinese competitive athletes within a centralized, high-pressure system, thereby extending leadership–morality frameworks beyond Western, educated, industrialized, rich, and democratic (WEIRD) and recreational contexts. It highlights how democratic and autocratic leadership styles operate in elite, performance-driven environments and provides a more context-sensitive basis for understanding leadership dynamics in competitive sport.

## 2. Theoretical framework and hypotheses

**Fig 1:**
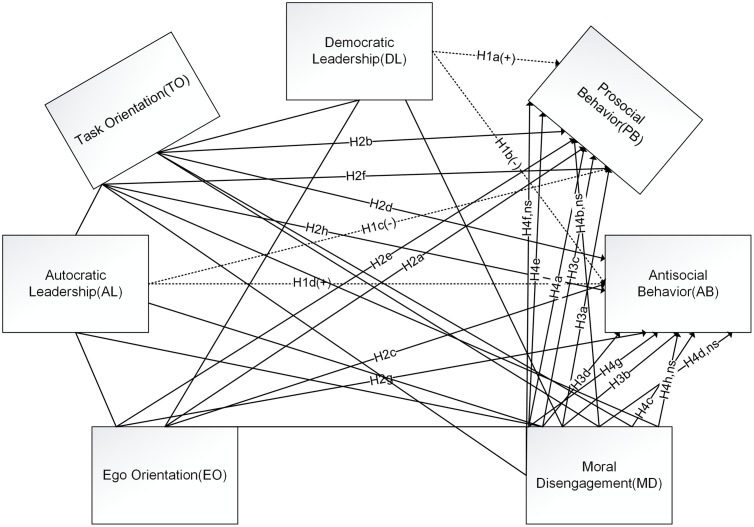
Proposed theoretical model. **Abbreviations:** DL = Democratic Leadership; AL = Autocratic Leadership; TO = Task Orientation; EO = Ego Orientation; MD = Moral Disengagement; PB = Prosocial Behavior; AB = Antisocial Behavior. Dashed arrows indicate direct effects (H1). Solid arrows indicate indirect (mediated) paths and sequential mediations (H2–H4). “+” and “–” mark the expected direction of the effect (positive/negative). “ns” marks paths that are hypothesized to be non-significant in the sequential chain (i.e., H4b, H4d, H4f, H4h via Task Orientation).

Guided by Social Cognitive Theory (SCT) and Achievement Goal Theory (AGT), we develop a dual-route framework in which coaching leadership shapes athletes’ moral conduct through two theoretically distinct but interrelated processes: a motivational route (task vs. ego orientation) and a moral-cognitive route (moral disengagement). These routes are expected to operate primarily in a complementary fashion, with each transmitting part of the leadership effect, but they may also diverge under high performance pressure when ego-involving goals coexist with attempts to justify norm violations. Accordingly, we model the motivational and moral-cognitive mechanisms simultaneously rather than in separate models, which allows us to estimate their unique and shared contributions to prosocial and antisocial behavior and to explore potential sequential links between them. Fig 1 depicts the proposed paths.

In this framework, the motivational and moral-cognitive routes are conceptually distinct: goal orientations primarily encode how success and failure are defined, whereas moral disengagement captures how athletes justify or neutralize norm violations. At the same time, within China’s centralized, performance-driven elite sport system, ego-involving goals and disengagement mechanisms are likely to co-occur under intense pressure, making it necessary to model the two routes simultaneously rather than assuming that one subsumes the other.

### 2.1 Direct effects

H1a. Democratic leadership → (+) Prosocial behavior.
H1b. Democratic leadership → (–) Antisocial behavior.
H1c. Autocratic leadership → (–) Prosocial behavior.
H1d. Autocratic leadership → (+) Antisocial behavior.

### 2.2 Mediation by goal orientation

H2a. Ego orientation mediates Democratic leadership → Prosocial behavior.
H2b. Task orientation mediates Democratic leadership → Prosocial behavior.
H2c. Ego orientation mediates Democratic leadership → Antisocial behavior.
H2d. Task orientation mediates Democratic leadership → Antisocial behavior.
H2e. Ego orientation mediates Autocratic leadership → Prosocial behavior.
H2f. Task orientation mediates Autocratic leadership → Prosocial behavior.
H2g. Ego orientation mediates Autocratic leadership → Antisocial behavior.
H2h. Task orientation mediates Autocratic leadership → Antisocial behavior.

### 2.3 Mediation by moral disengagement

H3a. Moral disengagement mediates Democratic leadership → Prosocial behavior.
H3b. Moral disengagement mediates Democratic leadership → Antisocial behavior.
H3c. Moral disengagement mediates Autocratic leadership → Prosocial behavior.
H3d. Moral disengagement mediates Autocratic leadership → Antisocial behavior.

### 2.4 Sequential mediation (goal orientation → moral disengagement)

H4a. Ego orientation → Moral disengagement sequentially mediates Democratic leadership → Prosocial behavior.
H4b. Task orientation → Moral disengagement does not sequentially mediate Democratic leadership → Prosocial behavior.
H4c. Ego orientation → Moral disengagement sequentially mediates Democratic leadership → Antisocial behavior.
H4d. Task orientation → Moral disengagement does not sequentially mediate Democratic leadership → Antisocial behavior.
H4e. Ego orientation → Moral disengagement sequentially mediates Autocratic leadership → Prosocial behavior.
H4f. Task orientation → Moral disengagement does not sequentially mediate Autocratic leadership → Prosocial behavior.
H4g. Ego orientation → Moral disengagement sequentially mediates Autocratic leadership → Antisocial behavior.
H4h. Task orientation → Moral disengagement does not sequentially mediate Autocratic leadership → Antisocial behavior.

## 3. Research participants and methods

### 3.1 Research participants

We used a cluster sampling strategy to recruit registered competitive athletes with official qualification at Level II or above under China’s National Athlete Technical Classification system (ascending grades: Level II, Level I, National Master Athlete [NMA], International Master Athlete [IMA]). Athletes were drawn from national, provincial, and municipal teams in Shandong, Henan, Anhui, Jiangsu, Zhejiang, Jilin, Shaanxi, Sichuan, Beijing, and Tianjin. A total of 1,382 questionnaires were distributed, and 1,239 valid responses were obtained (effective response rate = 89.7%).

Among the 1,239 respondents, 819 were male (66.1%) and 420 were female (33.9%). The average age was 20.10 years (SD = 2.54), and average training experience was 5.93 years (SD = 2.65). Sports included boxing, taekwondo, wrestling, discus throw, judo, gymnastics, volleyball, basketball, weightlifting, tennis, rugby, and canoeing. For descriptive purposes, these sports can be grouped into contact (boxing, taekwondo, wrestling, judo, rugby) versus non-contact sports (discus throw, gymnastics, volleyball, basketball, weightlifting, tennis, canoeing), and into individual (e.g., boxing, taekwondo, wrestling, discus throw, judo, gymnastics, weightlifting, tennis, canoeing) versus team sports (volleyball, basketball, rugby). The distribution across these categories was not fully balanced, as some individual and contact sports were more heavily represented than others. The study was not designed to yield a proportionally representative sample of all sport types. Regarding qualification, 693 were Level II (55.9%), 460 Level I (37.1%), and 86 National Master Athlete (6.9%) (no IMA cases in the present sample). Definition note: “Level II or above” refers to athletes officially certified by provincial or national sports authorities based on sport-specific performance standards, in ascending order: Level II → Level I → National Master Athlete → International Master Athlete.

This cluster-based strategy follows the administrative structure of Chinese elite sport and prioritizes ecological validity and access to intact training groups, while acknowledging that it does not constitute probability sampling of all registered athletes nationwide.

### 3.2 Measurement tools

All measurement tools used in this study were originally developed and validated in previous research. Prior to the main data collection, we conducted a small-scale pilot test with 50 competitive athletes who were not included in the final sample. The primary purpose of this pilot was to check item clarity, cultural appropriateness, and response formats; based on their feedback, minor wording refinements were made to several items to improve comprehensibility. Reliability analyses (Cronbach’s α) were then carried out using the full main sample. In the present data, Cronbach’s α values for all subscales exceeded.75, indicating acceptable to good internal consistency. Detailed α coefficients for each scale are reported in Sections 3.2.1–3.2.4.

#### 3.2.1 Coaching leadership behavior.

Coaching leadership was measured with the Leadership Scale for Sports (LSS), using the validated short form reported by Chiu et al. (2016) [27]; the Democratic and Autocratic subscales were used (5 items per subscale; 10 items total), with responses on a 5-point Likert scale (1 = never to 5 = always). Example items included “I encourage athletes to take part in decision-making” (Democratic) and “I instruct athletes in a commanding manner” (Autocratic). Internal consistency in the present sample was acceptable to excellent (Cronbach’s α = .90 and.85, respectively).

#### 3.2.2 Goal orientation.

Goal orientation was measured with the Task and Ego Orientation in Sport Questionnaire (TEOSQ) [28]. The questionnaire comprises 13 items tapping task (7 items) and ego (6 items) orientations. We made minor wording adaptations to fit the team-sport context (e.g., “classmates” → “teammates”), without altering item meaning. Responses were recorded on a 5-point scale (0 = not at all true to 4 = completely true). In this study, Cronbach’s α = .81 for ego and.89 for task.

#### 3.2.3 Moral disengagement.

We used the short version of the Sport Moral Disengagement Scale developed by Boardley et al. This single-factor scale includes 8 items (e.g., “It’s not wrong for an athlete to lie to a referee if it helps their team”), rated on a 7-point scale from 1 (strongly disagree) to 7 (strongly agree) [29]. Cronbach’s α was 0.77.

#### 3.2.4 Pro- and antisocial behavior.

Prosocial and antisocial behaviors were assessed with the Prosocial and Antisocial Behaviour in Sport Scale (PABSS) [30]. The instrument comprises 23 items forming four subscales—prosocial toward teammates (PT), prosocial toward opponents (PO), antisocial toward teammates (AT), and antisocial toward opponents (AO)—rated on a 5-point Likert scale (1 = never to 5 = very often). In the present sample, internal consistency was α = .83 (PT),.75 (PO),.85 (AT),.93 (AO); total α = .84.

#### 3.2.5 Control variables.

Several demographic and sport-related characteristics were included as control variables, given their established associations with athletes’ moral behavior. Specifically, gender (0 = female, 1 = male), age (years), and years of athletic training (years) were self-reported by participants. Competitive qualification level was coded as 1 = Level II, 2 = Level I, and 3 = National Master Athlete, based on athletes’ official registration records. These variables were entered as controls in all regression and SEM analyses to reduce potential confounding.

### 3.3 Statistical methods

Data were processed using SPSS 26.0 and AMOS 23.0. Analyses included reliability tests, tests for common method variance, descriptive statistics, ANOVA, correlation, and bootstrap-based mediation analyses. Structural Equation Modeling (SEM) was used to test the hypothesized mediation pathways. Gender, age, years of athletic training, and competitive qualification level were included as control variables in all models, Specifically:

Task orientation was modeled as a mediator between democratic leadership and prosocial behavior.

Ego orientation and moral disengagement were modeled as sequential mediators between autocratic leadership and antisocial behavior.

The model was tested using the maximum likelihood estimation method. Significance of indirect effects was verified using bias-corrected percentile bootstrap with 5,000 resamples, following Preacher et al.’s mediation test guidelines [31].

Before conducting regression analyses, assumptions of normality, linearity, and homoscedasticity were examined. The normality of residuals was assessed using skewness and kurtosis values, as well as visual inspection of histograms and Q-Q plots. Homoscedasticity was evaluated through the examination of scatterplots of residuals against predicted values. All assumptions were met, supporting the appropriateness of subsequent regression analyses.

To complement Harman’s single-factor test and further address potential common method variance (CMV), we adopted the unmeasured latent method construct (ULMC) technique in SEM. Specifically, a latent factor representing method variance was included in the measurement model, with all items loading on both their theoretical constructs and the latent method factor. The comparison of model fit indices (e.g., CFI, RMSEA) between the baseline and method-adjusted models indicated negligible improvement, suggesting that CMV was not a serious threat in this study. This dual-method approach strengthens the robustness of our findings by accounting for possible method bias beyond Harman’s test.

### 3.4 Data collection procedures

Data collection was conducted from April 2024 to January 2025. Written informed consent was obtained from all participants prior to their involvement in the study. For participants under the age of 18, additional written consent was obtained from their parents or legal guardians. Throughout the research process, participant anonymity and data confidentiality were strictly maintained in accordance with institutional ethical guidelines.

### 3.5 Ethical approval

This study was approved by the Sports Science Ethics Committee of Shandong Sport University (Approval Number: 2023030; approval date: (December 30, 2023). All experimental procedures strictly adhered to the ethical standards outlined in the Declaration of Helsinki, as well as the applicable laws, regulations, and ethical guidelines of China. Participant confidentiality and privacy were safeguarded throughout the research process.

## 4. Results and analysis

### 4.1 Common method bias

Because self-reported questionnaires were used, all data were derived from athletes’ own reports. To reduce the risk of common method bias, data were collected anonymously and additional procedural precautions were applied. Further, Harman’s single-factor test was conducted, as recommended in prior methodological research [32]. Specifically, all items for the study variables were loaded onto a single latent factor (single-factor model), and its fit indices were compared with those of the original seven-factor measurement model. The results showed that the seven-factor model (χ²/df = 3.38, RMSEA = 0.04, SRMR = 0.05, IFI = 0.91, CFI = 0.91, NFI = 0.88, TLI = 0.91) fit the data significantly better than the single-factor model (χ²/df = 14.70, RMSEA = 0.11, SRMR = 0.13, IFI = 0.47, CFI = 0.47, NFI = 0.46, TLI = 0.45), indicating that common method bias was not a serious issue in the present study.

### 4.2 Current status of athletes’ pro- and anti-social behaviors

The average total scores for pro-social and anti-social behaviors were 3.93 and 1.56, respectively (Table 1), suggesting that, on the whole, Chinese athletes demonstrated relatively high levels of pro-social behavior and relatively low levels of anti-social behavior. We also examined whether these behaviors varied based on gender or athletic qualification level.

**Table 1:** List of descriptive statistics and correlation analysis for each variable (n = 1239).

	X–	SD	1	2	3	4	5	6	7
DL	3.94	0.95	1.00						
AL	1.87	0.84	−0.38 ^***^	1.00					
EO	1.77	0.80	−0.21 ^***^	0.27 ^***^	1.00				
TO	3.10	0.75	0.53 ^***^	−0.22 ^***^	−0.15 ^***^	1.00			
MD	3.17	1.10	−0.19 ^***^	0.38^***^	0.34 ^***^	−0.13 ^***^	1.00		
PB	3.93	0.85	0.52 ^***^	−0.29 ^***^	−0.24 ^***^	0.58 ^***^	−0.21 ^***^	1.00	
AB	1.56	0.67	−0.31 ^***^	0.51 ^***^	0.28 ^***^	−0.26 ^***^	0.44^***^	−0.25 ^***^	1.00

**Note:**(*P < 0.05, **P < 0.01, **P < 0.001; the same notation applies below.), DL = Democratic Leadership; AL = Autocratic Leadership; TO = Task Orientation; EO = Ego Orientation; MD = Moral Disengagement; Sport Prosocial Behavior = PB; Sport Antisocial Behavior = AB.

For pro-social behavior, results indicated no significant main effect of gender (F = 0.06, P = 0.81) or qualification level (F = 0.38, P = 0.69), and no significant interaction effect (F = 1.37, P = 0.26).

For anti-social behavior, the main effect of gender was significant (F = 6.63, P < 0.05), whereas neither qualification level (F = 2.19, P = 0.11) nor the interaction between gender and qualification (F = 0.22, P = 0.80) reached statistical significance. Specifically, male athletes had a significantly higher mean score in anti-social behavior (X̄ = 1.59) compared to female athletes (X̄ = 1.49).

### 4.3 Correlation analysis among variables

Correlation analyses (Table 1) showed that democratic leadership, autocratic leadership, task orientation, ego orientation, sport moral disengagement, sport anti-social behavior, and sport pro-social behavior were significantly correlated with one another. All correlation coefficients were below 0.70, indicating no multicollinearity concerns. These findings lend support to subsequent tests of mediation effects.

To further enrich the descriptive profile of athletes’ moral conduct, we also examined differences in prosocial and antisocial behavior across age and training-duration groups. Athletes were divided into three age tertiles (≤19.1 years, 19.2–21.3 years, ≥ 21.4 years) and three groups based on years of training (≤4.7 years, 4.8–7.0 years, ≥ 7.1 years). One-way ANOVAs indicated no significant differences in prosocial or antisocial behavior across age groups (PB: F(2, 1236) = 0.31, p = .74; AB: F(2, 1236) = 0.01, p = .99) or across training-duration groups (PB: F(2, 1236) = 0.73, p = .48; AB: F(2, 1236) = 0.60, p = .55). Mean levels of prosocial behavior ranged from 3.91 to 3.97 and mean levels of antisocial behavior from 1.52 to 1.57 across all age and training groups, suggesting that moral conduct was relatively stable across age and experience bands within this elite sample.

### 4.4 Regression analysis of coaching leadership behaviors on athletes’ pro- and anti-social behaviors

Hierarchical regression analysis was employed to investigate the predictive relationships between coaching leadership behaviors and athletes’ pro- and anti-social behaviors (Tables 2 and 3). After controlling for demographic variables (gender, age, years of training, and athletic qualification), the results showed that:1. **Democratic leadership** significantly and positively predicted pro-social behavior (β = 0.52, P < 0.001) and significantly and negatively predicted anti-social behavior (β = −0.32, P < 0.001), explaining 28% and 11% of the variance, respectively.2. **Autocratic leadership** significantly and negatively predicted pro-social behavior (β = −0.30, P < 0.001) and significantly and positively predicted anti-social behavior (β = 0.51, P < 0.001), accounting for 9% and 26% of the variance, respectively.

**Table 2:** Regression analysis of democratic leadership behaviour on prosocial and antisocial behaviour in sport (n = 1239).

	Pro-Social Behavior		Anti-Social Behavior	
	*β*	*t*	*β*	*t*
Gender	−0.03	−1.21	−0.10	−3.61^***^
Age	0.04	1.82	−0.01	−0.38
Years of Training	−0.02	−0.70	−0.01	−0.44
Athletic Qualification	0.01	0.27	0.05	1.87
Democratic Leadership Behavior	0.52	21.60^***^	−0.32	−11.73^***^
F	94.88^***^		29.94^***^	
R^2^	0.28		0.11	
Adj R^2^	0.28		0.11	

**Table 3:** Regression analysis of autocratic leadership behaviour on prosocial and antisocial behaviour in sport (n = 1239).

	Pro-Social Behavior		Anti-Social Behavior	
	*β*	*t*	*β*	*t*
Gender	−0.05	−1.92	−0.09	−3.50^***^
Age	0.04	1.51	−0.02	−0.72
Years of Training	−0.02	−0.74	−0.02	−0.66
Athletic Qualification	0.03	1.01	0.01	0.27
Autocratic Leadership Behavior	−0.30	−10.80^***^	0.51	20.75^***^
F	24.61^***^		89.10^***^	
R^2^	0.09		0.27	
Adj R^2^	0.09		0.26	

**Note:** DL = Democratic Leadership; AL = Autocratic Leadership; TO = Task Orientation; EO = Ego Orientation; MD = Moral Disengagement; Sport Prosocial Behavior = PB; Sport Antisocial Behavior = AB.

Therefore, Hypotheses H1a, H1b, H1c, and H1d were all supported. These findings indicate that democratic leadership behaviors effectively promote athletes’ engagement in pro-social behavior and reduce anti-social behavior, whereas autocratic leadership behaviors tend to foster anti-social behavior and suppress pro-social behavior.

### 4.5 Testing the mediating effects of goal orientation and sport moral disengagement on the relationship between coaching leadership behaviors and pro-/anti-social behaviors

Hayes et al. noted in their study on mediation analyses that the bias-corrected percentile bootstrap method is superior to the Sobel test [33]. Specifically, the bootstrap method draws repeated samples from the original sample while keeping the probability of each observation being selected constant. In light of this, the SPSS 26.0 Macro Process 4.1 plug-in, compiled by Hayes, was employed to test the mediating effects [34]. The demographic variables (gender, age, years of training, and athletic qualification) were controlled in the mediation analyses. If the 95% confidence interval (CI) of the mediation effect contains 0, it indicates a non-significant mediation effect; otherwise, it indicates a significant mediation effect.

Regression analyses (Table 4) showed the following:1. **Democratic leadership behavior** significantly and negatively predicted ego orientation (β = −0.21, P < 0.001).2. When both democratic leadership behavior and ego orientation were entered into the model to predict sport moral disengagement, democratic leadership behavior still exhibited a significant negative predictive effect (β = −0.13, P < 0.001), whereas ego orientation had a significant positive predictive effect (β = 0.31, P < 0.001).3. When democratic leadership behavior, ego orientation, and sport moral disengagement were included simultaneously in predicting sport **pro-social behavior**, democratic leadership behavior significantly and positively predicted pro-social behavior (β = 0.48, P < 0.001), while ego orientation and sport moral disengagement both significantly and negatively predicted pro-social behavior (β = −0.11, P < 0.001; β = −0.08, P < 0.01).4. When democratic leadership behavior, ego orientation, and sport moral disengagement were entered together to predict sport **anti-social behavior**, democratic leadership behavior showed a significant negative predictive effect (β = −0.22, P < 0.001), whereas ego orientation and sport moral disengagement both significantly and positively predicted anti-social behavior (β = 0.11, P < 0.001; β = 0.36, P < 0.001).

**Table 4:** Regression analyses of tests of the mediating effects of ego orientation and sport moral disengagement on the relationship between democratic leadership behaviour and Prosocial and antisocial behaviour in sport (n = 1239).

Outcome	Model fit (R/ R²/ F)	Predictor	β(standardized)	t
Ego Orientation (EO)	0.22/ 0.05/ 12.29	DL	−0.21	−7.70***
Moral Disengagement (MD)	0.38/ 0.14/ 33.74	EO	0.31	11.29***
		DL	−0.13	−4.93***
Prosocial Behavior (PB)	0.55/ 0.30/ 75.80	EO	−0.11	−4.12***
		MD	−0.08	−3.24**
		DL	0.48	19.65***
Antisocial Behavior (AB)	0.52/ 0.27/ 63.68	EO	0.11	4.35***
		MD	0.36	13.47***
		DL	−0.22	−8.74***

**Note:** DL = Democratic Leadership; AL = Autocratic Leadership; TO = Task Orientation; EO = Ego Orientation; MD = Moral Disengagement; Sport Prosocial Behavior = PB; Sport Antisocial Behavior = AB.

These findings provide further evidence regarding the roles of ego orientation and sport moral disengagement as mediators in the relationship between democratic leadership and pro-/anti-social behaviors.

**Fig 2:**
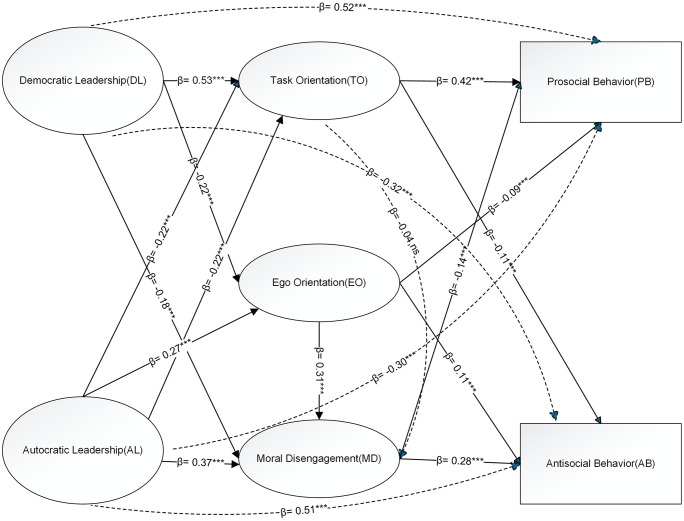
Structural model with standardized path coefficients (β). **Abbreviations:** DL  =  Democratic Leadership; AL  =  Autocratic Leadership; TO = Task Orientation; EO  =  Ego Orientation; MD  =  Moral Disengagement; PB  =  Prosocial Behavior; AB  =  Antisocial Behavior. Solid arrows represent significant indirect and sequential paths (H2–H4). Dashed arrows represent direct effects from leadership styles to outcomes (H1) and the non-significant link TO → MD. Signs of β indicate effect direction (positive/negative). ns  =  non-significant (p  ≥≥ .05). β  =  standardized path coefficient; significance codes: * p  << .05, ** p  << .01, *** p  << .001.

To visually illustrate the hypothesized mediation and path relations, the structural equation model (SEM) based on standardized estimates is presented in Fig 2.

Fig 2 presents the structural model linking coaching leadership to athletes’ prosocial and antisocial behavior via task/ego orientation and moral disengagement. Standardized path estimates are reported below (*** p < .001; n.s. = non-significant).

For the democratic-leadership block (Table 5), democratic leadership positively predicted task orientation (B = 0.53***), negatively predicted moral disengagement (B = −0.18***), and showed direct effects on prosocial (B = 0.28***) and antisocial behavior (B = −0.18***). Task orientation predicted higher prosocial behavior (B = 0.42***) and lower antisocial behavior (B = −0.11***), but did not significantly predict moral disengagement (B = −0.04, n.s.).

**Table 5:** Regression analyses of tests of the mediating effects of task orientation and sport moral disengagement on the relationship between democratic leadership behaviour and prosocial and antisocial behaviour in sport (n = 1239).

Outcome	Model fit (R/ R²/ F)	Predictor	β(standardized)	t
Task Orientation (TO)	0.53/ 0.28/ 94.03	DL	0.53	21.66***
Moral Disengagement (MD)	0.23/ 0.05/ 11.58	TO	−0.04	−1.17
		DL	−0.18	−5.48***
Prosocial Behavior (PB)	0.65/ 0.42/ 125.55	TO	0.42	16.24***
		MD	−0.10	−4.63***
		DL	0.28	10.98***
Antisocial Behavior (AB)	0.51/ 0.26/ 62.93	TO	−0.11	−3.87***
		MD	0.39	15.39***
		DL	−0.18	−6.20***

**Note:** DL = Democratic Leadership; AL = Autocratic Leadership; TO = Task Orientation; EO = Ego Orientation; MD = Moral Disengagement; Sport Prosocial Behavior = PB; Sport Antisocial Behavior = AB.

For the autocratic-leadership block (Table 6), autocratic leadership negatively predicted ego orientation (B = −0.22***) and positively predicted moral disengagement (B = 0.37***). It also showed direct effects on prosocial (B = −0.14***) and antisocial behavior (B = 0.37***). Ego orientation predicted higher prosocial behavior (B = 0.53***) and lower antisocial behavior (B = −0.14***), and did not significantly predict moral disengagement (B = −0.05, n.s.). Moral disengagement predicted lower prosocial behavior (B = −0.09***) and higher antisocial behavior (B = 0.28***).

**Table 6:** Overall model fit indices for the structural equation models examining the relationship between coaching style, goal orientation, and moral disengagement (n = 1239).

Outcome	Model fit (R/ R²/ F)	Predictor	β(standardized)	t
Ego Orientation (EO)	0.23/ 0.05/ 13.21	AL	−0.22	−8.06***
Moral Disengagement (MD)	0.40/ 0.16/ 37.95	EO	−0.05	−1.88
		AL	0.37	13.55***
Prosocial Behavior (PB)	0.61/ 0.38/ 105.96	EO	0.53	23.12***
		MD	−0.09	−3.72***
		AL	−0.14	−5.72***
Antisocial Behavior (AB)	0.59/ 0.35/ 96.11	EO	−0.14	−5.84***
		MD	0.28	11.20***
		AL	0.37	14.66***

**Note:** DL = Democratic Leadership; AL = Autocratic Leadership; TO = Task Orientation; EO = Ego Orientation; MD = Moral Disengagement; Sport Prosocial Behavior = PB; Sport Antisocial Behavior = AB.

These patterns support both independent (e.g., DL → TO → prosocial; AL → MD → antisocial) and sequential mediation pathways (e.g., AL → EO → MD → antisocial), consistent with the theoretical model.

The regression analysis results (Table 6) revealed that autocratic leadership behavior significantly and negatively predicted task orientation (β = −0.22, P < 0.001). When both autocratic leadership behavior and task orientation were included as predictors of sport moral disengagement, autocratic leadership behavior showed a significant positive predictive effect (β = 0.37, P < 0.001), while task orientation did not demonstrate a significant predictive effect (β = −0.05, P > 0.05). For instance, as shown in Table 7, democratic leadership explained 23.5% of the variance in athletes’ prosocial behavior (R² = 0.235). This indicates a moderate practical impact, suggesting that leadership style accounts for nearly one-quarter of the variability in athletes’ moral conduct, which is meaningful for real-world coaching practices.

**Table 7:** Regression coefficients and standard errors for predicting athletes’ moral behaviors from coaching style, goal orientation, and moral disengagement (n = 1239).

Outcome	Model fit (R/ R²/ F)	Predictor	β(standardized)	t
Ego Orientation (EO)	0.27/ 0.07/ 19.46	AL	0.27	9.75***
Moral Disengagement (MD)	0.46/ 0.21/ 55.30	EO	0.25	9.57***
		AL	0.31	11.73***
Prosocial Behavior (PB)	0.35/ 0.12/ 25.01	EO	−0.15	−5.23***
		MD	−0.08	−2.66**
		AL	−0.23	−7.72***
Antisocial Behavior (AB)	0.59/ 0.34/ 91.61	EO	0.09	3.62***
		MD	0.26	10.07***
		AL	0.38	15.13***

**Note:** DL = Democratic Leadership; AL = Autocratic Leadership; TO = Task Orientation; EO = Ego Orientation; MD = Moral Disengagement; Sport Prosocial Behavior = PB; Sport Antisocial Behavior = AB.

When autocratic leadership behavior, task orientation, and sport moral disengagement were simultaneously included as predictors of sport pro-social behavior, autocratic leadership behavior (β = −0.14, P < 0.001) and sport moral disengagement (β = −0.09, P < 0.001) exhibited significant negative predictive effects, whereas task orientation had a significant positive predictive effect (β = 0.53, P < 0.001).

Conversely, when predicting sport anti-social behavior, autocratic leadership behavior (β = 0.37, P < 0.001) and sport moral disengagement (β = 0.28, P < 0.001) demonstrated significant positive predictive effects, while task orientation had a significant negative predictive effect (β = −0.14, P < 0.001).

The structural model accounted for 42.3% of the variance in prosocial behavior and 39.7% in antisocial behavior (Table 8), indicating moderate explanatory power and practically relevant, though not exhaustive, coverage of the factors shaping athletes’ moral conduct.

**Table 8:** Bootstrap analyses for the mediated effects test (n = 1239).

Hypothesis	Pathway	Indirect Effect	LLCI	ULCI	Direct Effect	LLCI	ULCI	Result
H2a	DL → EO → PB	0.021	0.009	0.035	0.434	0.391	0.478	Supported
H3a	DL → MD → PB	0.017	0.007	0.029	—	—	—	Supported
H4a	DL → EO → MD → PB	0.005	0.001	0.010	—	—	—	Supported
H2b	DL→TO→PB	0.197	0.161	0.238	0.254	0.209	0.300	Supported
H4b	DL→TO→MD → PB	0.002	−0.002	0.006	—	—	—	Not Supported
H2c	DL → EO → AB	−0.017	−0.029	−0.008	−0.155	−0.190	−0.121	Supported
H3b	DL → MD → AB	−0.049	−0.074	−0.027	—	—	—	Supported
H4c	DL → EO → MD → AB	−0.016	−0.024	−0.010	—	—	—	Supported
H2d	DL→TO→AB	−0.041	−0.069	−0.014	−0.127	−0.167	−0.087	Supported
H4d	DL→TO→MD → AB	−0.005	−0.016	0.006	—	—	—	Not Supported
H2e	AL → EO → PB	−0.041	−0.061	−0.023	−0.237	−0.296	−0.179	Supported
H3c	AL → MD → PB	−0.025	−0.047	−0.004	—	—	—	Supported
H4e	AL → EO → MD → PB	−0.005	−0.011	−0.001	—	—	—	Supported
H2f	AL→TO→PB	−0.122	−0.166	−0.082	−0.151	−0.201	−0.102	Supported
H4f	AL→TO→MD → PB	−0.001	−0.003	0.001	—	—	—	Not Supported
H2g	AL → EO → AB	0.020	0.008	0.034	0.299	0.260	0.339	Supported
H3d	AL → MD → AB	0.083	0.057	0.112	—	—	—	Supported
H4g	AL → EO → MD → AB	0.014	0.009	0.021	—	—	—	Supported
H2h	AL→TO→AB	0.025	0.013	0.041	0.289	0.249	0.328	Supported
H4h	AL→TO→MD → AB	0.002	−0.001	0.006	—	—	—	Not Supported

**Note:** DL = Democratic Leadership; AL = Autocratic Leadership; TO = Task Orientation; EO = Ego Orientation; MD = Moral Disengagement; Sport Prosocial Behavior = PB; Sport Antisocial Behavior = AB.

For **democratic leadership → prosocial**, both mediator sets were supported. With **EO–MD** as mediators, the **indirect via EO = 0.021** (95% CI [0.009, 0.035]) and **via MD = 0.010** ([0.003, 0.020]) were significant, as was the **chain EO → MD = 0.005** ([0.001, 0.010]); the **direct effect = 0.434** ([0.391, 0.478]). Thus, **H2a, H3a, H4a** were supported. With **TO–MD** as mediators, **indirect via TO = 0.197** ([0.161, 0.238]) and **via MD = 0.017** ([0.007, 0.029]) were significant, whereas the **chain TO→MD = 0.002** ([−0.002, 0.006]) was not; the **direct effect = 0.254** ([0.209, 0.300]). Thus, **H2b** was supported and **H4b** was not.

For **democratic leadership → antisocial**, mediation also held in both specifications. With **EO–MD**, **indirect via EO = −0.017** ([−0.029, −0.008]) and **via MD = −0.033** ([−0.052, −0.017]) were significant, as was the **chain EO → MD = −0.016** ([−0.024, −0.010]); the **direct effect = −0.155** ([−0.190, −0.121]). Thus, **H2c, H3b, H4c** were supported. With **TO–MD**, **indirect via TO = −0.041** ([−0.069, −0.014]) and **via MD = −0.049** ([−0.074, −0.027]) were significant, whereas the **chain TO→MD = −0.005** ([−0.016, 0.006]) was not; the **direct effect = −0.127** ([−0.167, −0.087]). Thus, **H2d** was supported and **H4d** was not.

For **autocratic leadership → prosocial**, both mediator sets showed significant negative mediation. With **EO–MD**, **indirect via EO = −0.041** ([−0.061, −0.023]) and **via MD = −0.025** ([−0.047, −0.004]) were significant, as was the **chain EO → MD = −0.005** ([−0.011, −0.001]); the **direct effect = −0.237** ([−0.296, −0.179]). Thus, **H2e, H3c, H4e** were supported. With **TO–MD**, **indirect via TO = −0.122** ([−0.166, −0.082]) and **via MD = −0.034** ([−0.058, −0.012]) were significant, whereas the **chain TO→MD = −0.001** ([−0.003, 0.001]) was not; the **direct effect = −0.151** ([−0.201, −0.102]). Thus, **H2f** was supported and **H4f** was not.

For **autocratic leadership → antisocial**, the **TO–MD** model yielded significant positive indirect effects (**via TO = 0.025** [0.013, 0.041]; **via MD = 0.083** [0.057, 0.112]) and a non-significant **chain TO→MD = 0.002** ([−0.001, 0.006]); the **direct effect = 0.289** ([0.249, 0.328]). Thus, **H2h** was supported and **H4h** was not.

**Overall**, goal orientation (**EO/TO**) and **moral disengagement** provide robust **independent** mediation, while **chain mediation** emerges for **EO → MD** in several models (democratic→prosocial/antisocial; autocratic→prosocial) but not for **TO→MD**; significant direct paths remain, indicating **partial mediation** throughout.

Democratic leadership was positively associated with task orientation and negatively with both ego orientation and moral disengagement. Autocratic leadership had the reverse effect. These relationships are consistent with the applicability of ethical leadership frameworks in performance-driven sport settings.

## 5. Discussion

### 5.1 Summary of findings relative to hypotheses

Our study tested how coaching style relates to athletes’ moral conduct through goal orientations and moral disengagement. Results support all H1 direct effects: DL increases PB and reduces AB (H1a, H1b), whereas AL shows the opposite pattern (H1c, H1d).

For H2 single mediations, findings are partly supported: under DL, both EO and TO mediate effects on PB/AB (H2a–H2d supported); under AL, EO (but not TO) mediates PB/AB (H2e, H2g supported; H2f, H2h not supported).

For H3, MD acts as a mediator for DL → PB/AB and AL → PB/AB (H3a–H3d all supported).

For H4 sequential mediations, the EO → MD chain is robust: DL → EO → MD → PB/AB and AL → EO → MD → PB/AB are supported (H4a, H4c, H4e, H4g), whereas all TO→MD chains are not supported (H4b, H4d, H4f, H4h). Together, these results highlight two routes from leadership to moral conduct—motivational (EO/TO) and moral-cognitive (MD)—with EO → MD the most consistent pathway to AB (see Tables 4–7 and Fig 2).

### 5.2 Direct effects in context

Our findings show that democratic leadership (DL) corresponded to higher prosocial behavior (PB) and lower antisocial behavior (AB), whereas autocratic leadership (AL) showed the reverse pattern; prior work similarly indicates that involving athletes in decision-making reduces AB, while imposing unilateral decisions elevates AB [24].

From a social-cognitive perspective, coaches shape athletes’ behavioral standards through modeling, reinforcement, and climate setting, which helps to explain the direct links from leadership style to PB/AB observed here [35]. A well-known applied exemplar is Jackson’s democratic, voice-granting approach in professional basketball, where respectful communication and shared agency were used to cultivate cohesion and ethical norms, illustrating how DL can translate into harmonious relationships and prosocial team conduct [36].

Mechanistically, DL tends to cue task-focused meanings and dampen ego-focused meanings, whereas AL does the opposite; this motivational contrast foreshadows mediation via goal orientations that favor PB under DL and AB under AL [37].

### 5.3 Mediation via goal orientation

Goal orientation partly transmitted leadership effects: DL was associated with higher task orientation (TO) and lower ego orientation (EO), which aligned with more PB and less AB; by contrast, AL related to lower TO and higher EO with opposite behavioral consequences. In line with achievement-goal accounts, TO emphasizes mastery and cooperation that are compatible with PB, whereas EO emphasizes normative comparison that can escalate competitive hostility and moral compromise, thereby increasing AB.

### 5.4 Mediation via moral disengagement

Moral disengagement (MD) served as a proximal cognitive mechanism linking leadership to behavior. DL related to lower MD and, in turn, more PB and less AB; AL related to higher MD with the opposite pattern. Conceptually, democratic, participative routines reduce the need for justificatory distortions (e.g., diffusion of responsibility), whereas controlling routines heighten stress and make disengagement rationalizations more accessible—thereby channeling effects toward AB.

### 5.5 Sequential mediation and broader comparators

The sequential chain EO → MD → AB emerged as the most consistent pathway, particularly under AL, whereas TO-based chains were weaker in this elite, outcome-salient setting. This pattern is consistent with management evidence that participative leadership enhances rule adherence and ethical choice under pressure, offering an external benchmark for why DL climates constrain disengagement-driven misconduct [38]. In the Chinese elite-sport context, where selection, promotion, and resource allocation are tightly coupled to competitive outcomes, ego-involving meanings and moral disengagement may be especially prone to reinforcing one another rather than operating in isolation. This helps to explain why EO-based sequential chains are more robust than TO-based chains in our data and suggests that motivational and moral-cognitive routes are complementary under sustained performance pressure. Complementarily, work on authoritarian/controlling leadership documents greater disengagement, trust erosion, and ethical lapses in high-stakes organizational systems, paralleling the AL-linked sequence we observe in sport [39].

At the same time, the pattern of mediation observed here must be interpreted in light of the study’s exclusive reliance on self-report questionnaires. Although our procedural steps and common-method checks (including Harman’s test and a latent method factor) suggest that common-method variance is unlikely to fully account for the findings, shared-source measurement may still inflate associations among conceptually related constructs, particularly those involving moral disengagement. For example, athletes who are more willing to acknowledge antisocial tendencies may also report higher disengagement and ego orientation, which could accentuate the EO–MD–AB chain. Future studies employing multi-informant designs (e.g., coach or teammate reports) and behavioral indicators would be better positioned to test the robustness of these mediation patterns and to distinguish substantive processes from reporting style.

### 5.6 Theoretical implications

The study integrates leadership style (DL/AL), goal orientations (EO/TO), and moral disengagement (MD) into a dual-route account of moral behavior, addressing prior work that typically modeled motivational and moral-cognitive pathways separately and clarifying how goal orientations intersect with moral self-regulation within a single framework [40,29]. Culturally, validating this dual-route framework in a high-performance Chinese context extends leadership–morality findings beyond Western samples and, together with sport-specific research on Chinese athletes, underscores the importance of coach-created climates for understanding these processes [41,42].

Conceptually, pinpointing the comparative strength of the EO → MD → AB path—alongside the weaker sequential role of TO for prosocial behavior in elite settings—clarifies when and why ego-involving climates are more likely to translate into misconduct and outlines a focused agenda for future tests of leadership-to-morality processes in sport [3].

The findings not only confirm the existence of two distinct pathways but also shed light on their interplay. The strong connection between ego-orientation, moral disengagement, and antisocial behavior suggests that motivational and moral-cognitive processes often reinforce each other in this context—ego-driven goals tend to fuel moral disengagement rather than counteract it. Meanwhile, the weaker indirect effects observed through task orientation indicate that a mastery-focused motivational climate may encourage prosocial behavior without significantly altering moral-cognitive processing. This overall pattern supports the idea that leadership shapes moral conduct through multiple, complementary mechanisms while acknowledging that motivational and moral-cognitive processes might diverge under different circumstances. While the current study wasn’t designed to formally test interactions between these pathways, the dual-route framework highlights several promising avenues for future research. For instance, moral disengagement could act as a moderator, strengthening the link between ego orientation and antisocial behavior when moral disengagement levels are high. On the other hand, certain leadership behaviors might either weaken or intensify how ego-driven goals translate into moral disengagement, thereby shifting the balance between these pathways. To explore these dynamics more thoroughly, future studies could employ longitudinal or experimental designs that manipulate leadership climates and monitor changes in both goal orientations and moral disengagement over time.

### 5.7 Practical implications

The findings offer several practical implications for coach education and sport governance, particularly in performance-driven systems like Chinese elite sport [43]. Coach education programs should prioritize democratic, athlete-centered practices—such as participative decision-making, value-based feedback, and transparent communication—as these approaches are linked to higher prosocial behavior and lower antisocial tendencies. Organizations can further support ethical development by structuring training environments and evaluation systems to reward mastery, cooperation, and ethical conduct alongside competitive outcomes, thereby reducing an overemphasis on ego-driven goals that may encourage disengagement and misconduct [44]. Additionally, since both motivational and moral-cognitive pathways influence behavior, interventions should address not only athletes’ goal orientations but also the justifications that normalize rule-breaking, such as diffusing responsibility or downplaying harm. However, as the current evidence is cross-sectional, these recommendations should be viewed as preliminary rather than definitive, requiring further validation through longitudinal and experimental studies to determine whether shifts in leadership practices lead to measurable changes in athletes’ goal orientations, moral disengagement, and behavior [45].

## 6. Conclusion

This study shows that coaching leadership styles are systematically linked to athletes’ moral behavior. Democratic leadership promotes prosocial behavior and reduces antisocial behavior, whereas autocratic leadership exhibits the opposite pattern. These effects operate not only directly but also indirectly through motivational (task vs. ego orientation) and moral-cognitive (moral disengagement) pathways. Specifically, democratic leadership suppresses ego orientation and moral disengagement and, together with task orientation, supports prosocial conduct; autocratic leadership heightens ego orientation and moral disengagement, thereby elevating antisocial tendencies. Taken together, the model clarifies how leadership effects propagate through distinct yet complementary mechanisms and provides an explanatory basis for ethical climate building in competitive sport.

### 6.1 Limitations

This study has several limitations that should be considered when interpreting the findings. First, the research used a cross-sectional design with data collected from a single source through self-reports, which may introduce issues such as social desirability bias and common-method variance. Although steps were taken to address these concerns—including ensuring participant anonymity, counterbalancing survey items, and conducting statistical checks like single-factor analysis—these risks remain. Second, the sample was drawn exclusively from competitive sports in China, meaning that the unique cultural and structural aspects of this system may limit how applicable the results are to other countries, different levels of sports participation, or recreational settings. Additionally, the study did not test whether the measurement tools performed consistently across various subgroups. Third, all constructs were measured using adapted self-report scales, and the lack of behavioral data, assessments from multiple sources (such as coaches or teammates), or observational information may affect the validity of the findings. Fourth, the analytical model did not explore alternative explanations for the observed processes—for example, whether factors like moral identity or team climate might influence the results through moderated mediation. The use of single-level analysis might also overlook effects related to team-level climate, and there may be limited statistical power to detect subtle differences between specific pathways, such as comparing task-related to ego-related disengagement. These limitations affect the ability to draw causal conclusions and to generalize the findings, highlighting the need to interpret the results within the context of these constraints.

In addition, the distribution of athletes across sport types (e.g., contact vs. non-contact, individual vs. team sports) was not fully balanced, with some categories more heavily represented than others. This imbalance may limit the generalizability of the findings to underrepresented sport types and suggests that future research should employ stratified sampling or targeted recruitment to achieve more proportionate coverage across sport categories.

### 6.2 Future directions

Future research should incorporate multi-source and behavioral measures (e.g., peer/coach ratings, observational coding) to triangulate self-reports and reduce common-method concerns. Longitudinal and experimental designs are recommended to identify temporal dynamics and test causal mechanisms linking leadership, motivation, moral disengagement, and behavior. Cross-cultural comparisons—ideally with tests of measurement invariance—could assess the portability of the model and potential moderation by sociocultural factors. Finally, expanding the model to include additional social-contextual mechanisms (e.g., coach–athlete relationship quality, team norms/communication) and multilevel structures (team- and organization-level leadership climates) may further refine explanatory power.

## Supporting information

S1 File Raw dataset used for all statistical analyses. This file contains participant-level data including demographic variables (gender, age, years of training), coach leadership style scores (democratic, autocratic), psychological mediators (task orientation, ego orientation, moral disengagement), and outcome variables (prosocial and antisocial behaviors). All variables are anonymized and scaled.
(CSV)
